# A capsaicinoid-based soft drug, AG1529, for attenuating TRPV1-mediated histaminergic and inflammatory sensory neuron excitability

**DOI:** 10.1038/s41598-020-80725-z

**Published:** 2021-01-08

**Authors:** Magdalena Nikolaeva-Koleva, Laura Butron, Sara González-Rodríguez, Isabel Devesa, Pierluigi Valente, Marta Serafini, Armando A. Genazzani, Tracey Pirali, Gregorio Fernández Ballester, Asia Fernández-Carvajal, Antonio Ferrer-Montiel

**Affiliations:** 1 Instituto de Investigación, Desarrollo e Innovación en Biotecnología Sanitaria de Elche (IDiBE), Universitas Miguel Hernández, 03202 Elche, Spain; 2 AntalGenics SL, Ed. Quorum III, UMH Scientific Park, 03202 Elche, Spain; 3 Department of Experimental Medicine, Section of Physiology, University of Genova, Viale Benedetto XV 3, 16132 Genoa, Italy; 4 Dipartimento Di Scienze del Farmaco, Università Degli Studi del Piemonte Orientale, 28100 Novara, Italy; 5 Present Address: Laboratorio de Farmacología, Facultad de Medicina, Instituto Universitario de Oncología del Principado de Asturias (IUOPA), Universidad de Oviedo, 33006 Oviedo, Spain

**Keywords:** Ion channels in the nervous system, Receptor pharmacology

## Abstract

TRPV1, a member of the transient receptor potential (TRP) family, is a nonselective calcium permeable ion channel gated by physical and chemical stimuli. In the skin, TRPV1 plays an important role in neurogenic inflammation, pain and pruritus associated to many dermatological diseases. Consequently, TRPV1 modulators could represent pharmacological tools to respond to important patient needs that still represent an unmet medical demand. Previously, we reported the design of capsaicinoid-based molecules that undergo dermal deactivation (soft drugs), thus preventing their long-term dermal accumulation. Here, we investigated the pharmacological properties of the lead antagonist, 2-((4-hydroxy-2-iodo-5-methoxybenzyl) amino)-2-oxoethyl dodecanoate (AG1529), on heterologously expressed human TRPV1 (hTRPV1), on nociceptor excitability and on an in vivo model of acute pruritus. We report that AG1529 competitively blocked capsaicin-evoked activation of hTRPV1 with micromolar potency, moderately affected pH-induced gating, and did not alter voltage- and heat-mediated responses. AG1529 displays modest receptor selectivity as it mildly blocked recombinant hTRPA1 and hTRPM8 channels. In primary cultures of rat dorsal root ganglion (DRG) neurons, AG1529 potently reduced capsaicin-evoked neuronal firing. AG1529 exhibited lower potency on pH-evoked TRPV1 firing, and TRPA1-elicited nociceptor excitability. Furthermore, AG1529 abolished histaminergic and inflammation mediated TRPV1 sensitization in primary cultures of DRG neurons. Noteworthy, dermal wiping of AG1529, either in an acetone-based formulation or in an anhydrous ointment, dose-dependently attenuated acute histaminergic itch in a rodent model. This cutaneous anti-pruritic effect was devoid of the normal nocifensive action evoked by the burning sensation of capsaicin. Taken together, these preclinical results unveil the mode of action of AG1529 on TRPV1 channels and substantiate the tenet that this capsaicinoid-based soft drug is a promising candidate for drug development as a topical anti-pruritic and anti-inflammatory medication.

## Introduction

Transient receptor potential vanilloid channel (TRPV1) is a thermally activated ion channel that plays a pivotal role in thermosensation, particularly in the detection of environmental noxious temperatures (> 42 °C)^1,2^. Apart from heat, TRPV1 channels are also activated by molecules containing a vanilloid group, endovanilloid/endocannabinoid molecules such as anandamide, extracellular acidic pH and voltage depolarization^3–9^. Although TRPV1 is highly expressed in the neuronal system, it is also widely present in the skin, mainly in the peripheral terminals of nociceptors, keratinocytes, sebocytes and immune cells^10–15^. Cutaneous TRPV1 channels have been primarily involved in temperature sensing, modulating skin barrier function, and in neurogenic inflammation, pain an pruritus^13,16^.

Notably, cutaneous TRPV1 channels are end targets for inflammatory and pruritogenic agents^17–19^. These agents activate intracellular signalling pathways that converge onto TRPV1, increasing its gating^20^ and/or promoting the surface expression of channels^21^. Algesically-sensitized TRPV1 channels notably increase the excitability of nociceptors that lead to pain and pruritic signals^22^. Consequently, cumulative evidence indicates that the TRPV1 channel is a valid pharmacological target to treat peripheral inflammatory pain as well as pruritus^23^. Indeed, the desensitizing action of capsaicin on TRPV1 has been long used clinically to treat cutaneous inflammatory conditions, including inflammation, pain and pruritus^24^. However, despite its good therapeutic action, the burning sensation associated to capsaicin, due to its agonistic activity, decreases patient adherence to the treatment. In addition, some reports suggest that its long-term dermal accumulation due to its poor elimination may induce dermal carcinogenesis that could be potentiated by UV exposure^25,26^. Thus, there is a need to develop TRPV1 modulators that, by preserving the therapeutic efficacy of the vanilloid molecule, eliminate the burning sensation and do not accumulate in the skin. Accordingly, several potent TRPV1 antagonists have been developed in recent years and tested in animal models and clinical trials^27^. Unfortunately, hyperthermia, due to the systemic absorption of the antagonists, has prevented many of these compounds to reaching the clinic^28^. Nonetheless, there are modulators, such as N-[(1R)-1-[3,5-difluoro-4-[(methylsulfonyl)amino]phenyl]ethyl]-3-[2-propyl-6-(trifluoromethyl)-3-pyridinyl]-, (2E)- (PAC-14028, Asivatrep), that have shown anti-inflammatory and anti-pruritic activities in phase II clinical trials, displaying an acceptable pharmacological profile^29,30^.

To increase the armamentarium of TRPV1 antagonists with enhanced therapeutic index, we previously introduced the concept of soft drugs to the capsaicinoid family, i.e. drugs that suffer dermal and systemic deactivation after exerting their activity^31^. Thus, capsaicinoid-based soft drugs do not show any increased risk associated with skin accumulation and any side effect related to systemic exposure. We designed TRPV1 antagonists that blocked the rodent TRPV1 with micromolar potency and were sensitive to deactivation by dermal esterases^31^. Our capsaicinoid antagonists preserve the pharmacological scaffold of the vanilloid group, but are devoid of the burning sensation associated with capsaicin. Compound AG1529 was selected as a promising candidate for further development due to its in vivo therapeutic activity in animal models of inflammatory pain and pruritus^31^. Here, we further characterize the activity of AG1529 and show that this capsaicinoid blocks the human TRPV1 ortholog (hTRPV1) with micromolar potency. Our data suggest that AG1529 acts as a competitive capsaicin antagonist. This compound modestly blocks pH-evoked hTRPV1 gating but not voltage and temperature activation. It shows modest receptor selectivity as it moderately blocks recombinant hTRPA1 and hTRPM8 channels. Furthermore, AG1529 reduces capsaicin-induced sensory neuron excitability, and attenuates histamine- and inflammatory-sensitization of TRPV1 in primary DRG cultures. Notably, a dermal formulation of AG1529 reduces the itch produced by local injection of histamine in rats. Taken together, our preclinical results suggest that AG1529 is a good candidate for development as an anti-inflammatory and anti-pruritic drug associated to cutaneous disorders.

## Results

### AG1529 reversibly blocks hTRPV1 with micromolar potency

**Figure 1:**
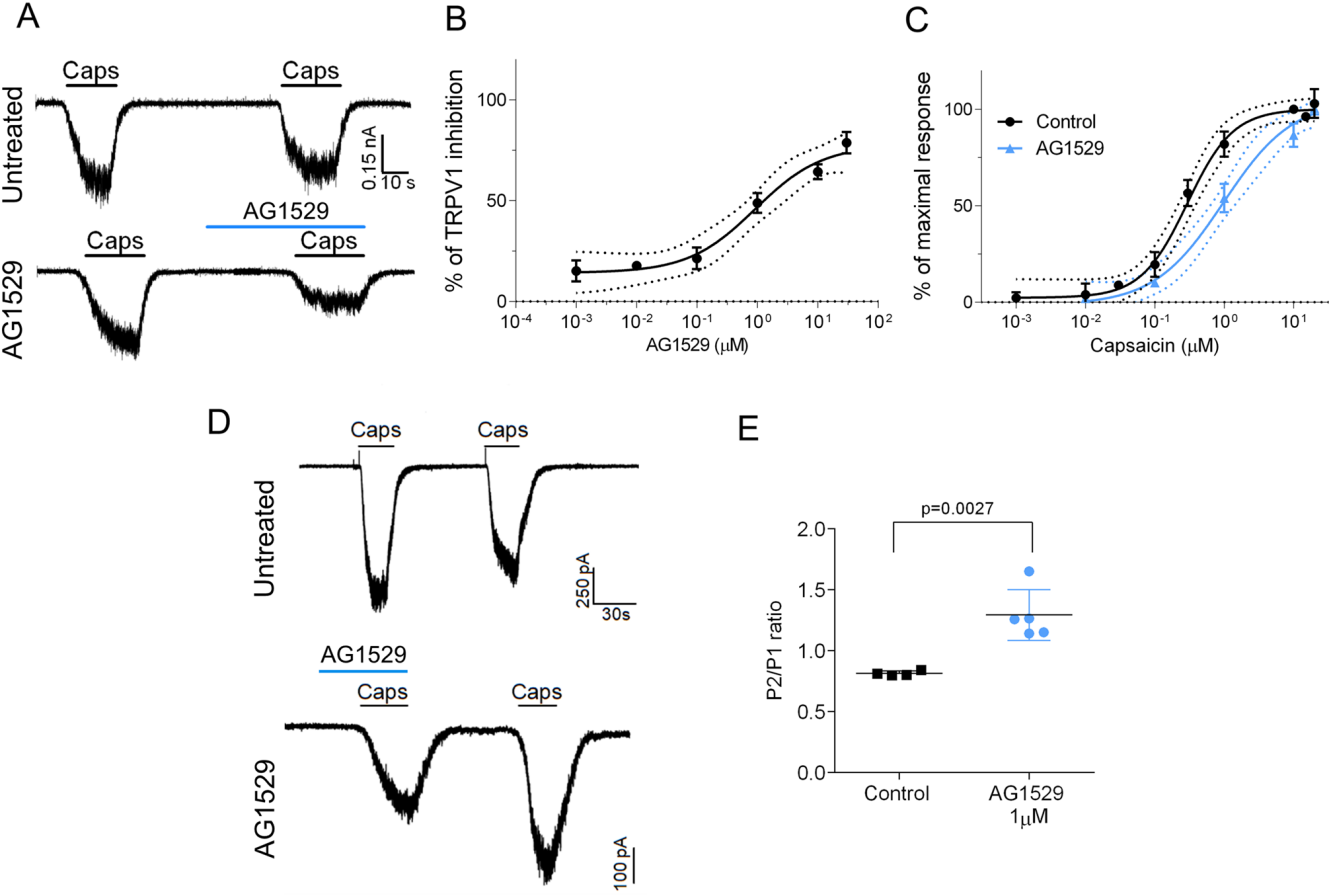
AG1529 reversibly blocks capsaicin activation of hTRPV1. Electrophysiological evaluation of AG1529 on heterologously expressed hTRPV1 channel. (**A**) Representative capsaicin-evoked hTRPV1 current recorded at a holding potential of − 60 mV for: (i) control condition (Untreated cells), where cells were exposed to two capsaicin pulses (0.5 μM), interspersed by a washing period; and, (ii) Treated condition (AG1529 cells), where cells were exposed to a capsaicin pulse (0.5 μM), and after a washing period, cells were exposed to 1 µM AG1529 for 30 s before the second 0.5 μM capsaicin pulse. The percentage of TRPV1 inhibition was calculated as: (% inhibition) = [1 − {(I_cap1 _− I_cap2_)_AG1529_/(I_cap1 _− I_cap2_)_Control_}] × 100; (I_cap1 _− I_cap2_)_Control_ accounts for the extend of TRPV1-induced desensitization by capsaicin (control). (**B**) Dose–response curve of AG1529 were fitted to a Michaelis–Menten Isotherm. The best fit provided an IC_50_ value of 0.92 (95% CI 0.28–3.01) µM and Hill coefficient of n_H_ 1.31 (95% CI 0.81–1.82) (n = 32). (**C**) Capsaicin dose response in the absence (black) and presence of AG1549 (blue). Data were fitted to a Michaelis–Menten Isotherm. The best fitted values for capsaicin EC_50_ value was 0.29 (95% CI 0.20–0.35) µM (n = 29) in the absence of 1 µM AG1529 and 0.98 (95% CI 0.6–1.13) µM in its presence (blue curve, n = 21). Hill coefficient in the absence of AG1529 was 1.3 (0.9–1.6) and in its presence was 1.00 (95% CI 0.7–1.2). (**D**) Representative repetitive capsaicin-elicited hTRPV1 currents recordings at − 60 mV holding potential in the absence (Untreated cells) and presence of 1 µM AG1529 before the first vanilloid pulse (AG1529). (**E**) P2/P1 ratio denoting TRPV1 current evoked by each 0.5 μM capsaicin pulse, normalized to first vanilloid pulse, in the absence (control, n = 4) and the presence of 1 µM AG1529 (n = 5) before the first capsaicin pulse. Horizontal lines denote the duration of compound pulse. All data are expressed as mean ± SEM. Data was analysed using an unpaired, two-tail Student’s t-test. p-value is indicated.

To assess the therapeutic potential of AG1529, we tested its inhibitory activity on recombinantly expressed hTRPV1. As depicted in Fig. 1A, capsaicin-evoked inward currents were reduced ≥ 60% by the concomitant administration of 1 μM AG1529 to HEK293 cells expressing hTRPV1. A dose–response curve revealed an IC_50_ of 0.9 ± 0.5 μM (n = 32) and a Hill coefficient n_H_ = 0.8 ± 0.4 for AG1529 blocking hTRPV1 channel activity (Fig. 1B). Note that we could only reach 78% blockade efficacy due to the water insolubility of AG1529 at > 30 μM. Higher compound concentrations required the use of high DMSO (> 1%) that affected the activity measurements.

We next analyzed the effect of AG1529 on the capsaicin potency activating hTRPV1 channels. For this experiment, we compared the capsaicin potency activating hTRPV1 in the absence and presence of 1 μM of AG1529 that corresponds to its IC_50_ value. At this AG1529 concentration, sufficient capsaicin-evoked current remains to be reliably quantified. Figure 1C shows that the capsaicin dose–response for hTRPV1 activation was significantly right-shifted to higher agonist concentrations in the presence of 1 μM AG1529 without affecting the capsaicin efficacy. Fitting the experimental data to a Michaelis–Menten isotherm curve revealed a shift of the vanilloid EC_50_ from 0.28 ± 0.03 μM to 0.9 ± 0.1 μM (i.e. threefold increase, p < 0.0001, unpaired, two-tail, t-Student, n = 29), and a decrease of the agonist Hill coefficient (n_H_) from 1.3 ± 0.2 to 1.0 ± 0.1, p < 0.0001, unpaired, two-tail t-Student). This displacement of the capsaicin potency (EC_50_) activating TRPV1 to a higher vanilloid concentration in the presence of AG1529 is consistent with a competitive inhibitory mechanism.

To further evaluate the activity of AG1529, we next investigated if its TRPV1 inhibitory activity was reversible. For this experiment, we used a paradigm consisting on two capsaicin pulses interspersed by a washing period (Fig. 1D). We quantified the ratio P2/P1 as an indicator of capsaicin response recovery. Compound AG1529 at 1 μM was perfused during the first agonist pulse to block 50% of the ionic current (Fig. 1D, bottom panel). As depicted in panel D (top traces), repeated application of capsaicin desensitized hTRPV1 channels, as evidenced by the lower percentage of current activated by the second (P2) capsaicin pulse (Fig. 1E, P2/P1 = 0.81 ± 0.01, n = 4). In contrast, the same paradigm applied to cells that were exposed to 1 μM AG1529 during the first capsaicin pulse, resulted in an increment of the second capsaicin response (Fig. 1D, bottom trace; and Fig. 1E; P2/P1 = 1.30 ± 0.09 (n = 5), p = 0.0027, AG1529 vs. control, unpaired, two-tail, t-student). These data are consistent with: (i) a reversible blockade of hTRPV1 by AG1529; and, (ii) a partial inhibition of capsaicin-induced TRPV1 tachyphylaxis by AG1519. Taken together, these findings indicate that AG1529 is a reversible blocker, and further suggest that it acts as a competitive capsaicin antagonist of hTRPV1.

### AG1529 modestly affects other modalities of hTRPV1 gating

**Figure 2:**
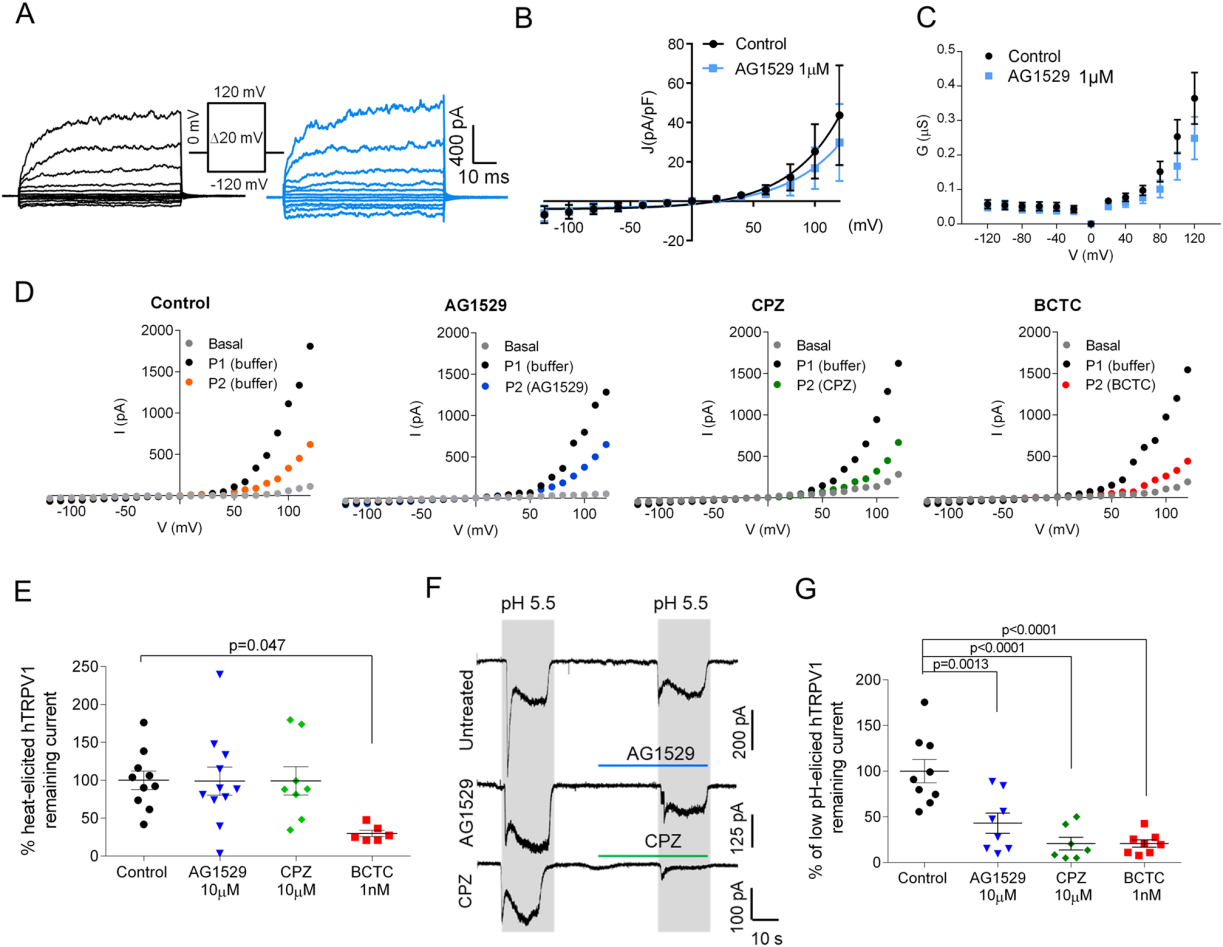
AG1529 modestly affects pH, voltage and heat activation. (**A**) Representative hTRPV1 current recordings elicited by voltage steps protocol from − 120 mV to 120 mV in 100 ms steps of 20 mV from a holding potential of 0 mV. Black traces represent currents evoked in the absence of AG1529, and blue traces in the presence of 1 µM AG1529. (**B**) Current density to voltage relationship for ionic currents in the absence (n = 8) and presence of the antagonist (n = 8). (**C**) Conductance to voltage relationship for the ionic currents in the absence and presence of the antagonist. Conductance values were estimated from the current density values, using as V_r_ the x-intercept of the ionic current. (**D**) Representative I-V relationships of hTRPV1 ionic currents at 43 °C in the absence (control) and presence of 10 µM AG1529 (n = 11) or 10 µM capsazepine (CPZ, n = 7) or 1 nM BCTC (n = 6). Basal, denotes the ionic currents activated at 37 °C; P1 and P2, the ionic currents evoked at 43 °C in the absence and presence of the compounds, respectively. For control conditions, P2 was evoked in the absence of compounds (buffer) denoting the desensitization induced by heat and voltage. Ionic currents were recorded with a voltage ramp protocol from − 120 mV to 120 mV in 300 ms**.** Compounds were applied at 34 °C during 30 s before the second temperature increase to 43 °C. (**E**) Normalized voltage-evoked ionic current at 43 °C and + 120 mV, in the absence (control) and presence of the antagonists. Remaining TRPV1 current after P2 (desensitization) was used for normalization. Data are expressed as mean ± SEM, and were analysed using the One-Way Anova (F(3,31) = 3.5, p = 0.0269) followed by the Bonferroni´s post-hoc test, p-value for statistical difference is indicated. (**F**) Representative acid pH-elicited hTRPV1 ionic currents evoked by two pH 5.5 pulses in control cells (Untreated), and cells exposed to 10 µM AG1529 (AG1529) or 10 µM capsazepine (CPZ) for 30 s before the second pH pulse. Amiloride (50 µM) was used in all buffers to block endogenous ASIC channels expressed by cells. Currents were elicited at a holding V = -60 mV. (**G**) Normalized hTRPV1 pH-evoked inward currents elicited for control (n =9 ), 10 µM AG1529 (n = 8), 10 µM capsazepine (n = 7) and 1 nM BCTC (n =8 ). Normalized responses were estimated as (I_pH2_/I_pH1_) × 100. Grey rectangles denote the duration of the pH pulses and horizontal lines the duration of the antagonists pulses. All data is expressed as mean ± SEM. Data were analysed using the One-Way Anova (F(3,28) = 15.5, p < 0.0001) followed by the Bonferroni´s post-hoc test, p-values for statistical difference are indicated.

Next, we investigated whether AG1529 also inhibited hTRPV1 activity triggered by other activating stimuli, such as voltage, temperature (43 °C) and acidic pH (pH 5.5). Figure 2A illustrates the effect of AG1529 on voltage-induced activation of hTRPV1 gating at 22 °C. Although at room temperature TRPV1 channels display a low sensitivity to voltage stimulation, they can be opened at depolarized voltages ≥ 50 mV (Fig. 2A), giving rise to outward currents. Voltage gating of hTRPV1 was not significantly altered by the presence of 1 μM AG1529 (Fig. 2B). The current density-to-voltage relationship depicts a non-significant inhibitory effect, barely discernible at potentials > 100 mV (Fig. 2B). A conductance-to-voltage relationship further indicates a lack of effect of AG1529 on hTRPV1 voltage-evoked currents (Fig. 2C).

To study the effect on temperature activation, we recorded I–V curves at 43 °C in the absence and presence of 10 μM AG1529. We increased the compound concentration considering the lack of effect seen at 1 μM. As controls, we used the well-known TRPV1 antagonists *N*-[2-(4-Chlorophenyl)ethyl]-7,8-dihydroxy-1,3,4,5-tetrahydro-2*H*-2- benzazepine-2-carbothioamide (Capsazepine (CPZ), 10 μM) and 4-(3-Chloro-2-pyridinyl)-N-[4-(1,1-dimethylethyl)phenyl]-1-piperazinecarboxamide (BCTC, 1 nM). Representatives ramps of currents evoked at 43 °C are shown in Fig. 2D. As seen, in control cells voltage ramps activated large ionic outward currents (P1) that partially desensitized when a second ramp was recorded 30 s later (P2). Similar recordings were obtained when the antagonists were present during the second voltage ramp (Fig. 2D). Quantitation of the ionic current evoked at 43 °C and + 120 mV in the absence and presence of the compounds reveals that only BCTC at 1 nM significantly blocked the evoked currents (Fig. 2E, F(3,31) = 3.5, p = 0.027, and p = 0.0473 for control vs. BCTC, Bonferroni´s post-hoc test). Note that AG1529 and capsazepine were both inactive.

Last, we evaluated whether AG1529 affected hTRPV1 gating evoked by acidic extracellular pH. For this experiment, we elicited ionic currents in hTRPV1-HEK293 cells with two pulses of extracellular buffer at pH 5.5, interspersed by a washing step (Fig. 2F). pH-activated ionic currents displayed a fast inactivating component due to activation of ASIC channels, followed by a stationary phase that corresponds to hTRPV1 responses (Fig. 2F). The fast component was blocked by 50 μM amiloride (Fig. 2F). Figure 2F shows that AG1529 and capsazepine significantly reduced the pH-gated ionic currents. Quantitation of this inhibitory activity reveals that 10 μM AG1529 reduced by 50% pH-activated ionic currents, akin to 10 μM capsazepine and 1 nM BCTC (Fig. 2G, F(3,28) = 15.50, p < 0.0001; p = 0.0013 AG1529 vs. control; and p < 0.0001 CPZ and BCTC vs. control, Bonferroni´s post-hoc test). No statistical differences were observed between the AG1529 and CPZ blocking pH-evoked activity. Thus, our results indicate that AG1529 moderately blocks pH-induced hTRPV1 activity, without significantly affecting voltage- and heat-elicited responses.

### AG1529 moderately modulates TRPM8 and TRPA1 channel activity

**Figure 3:**
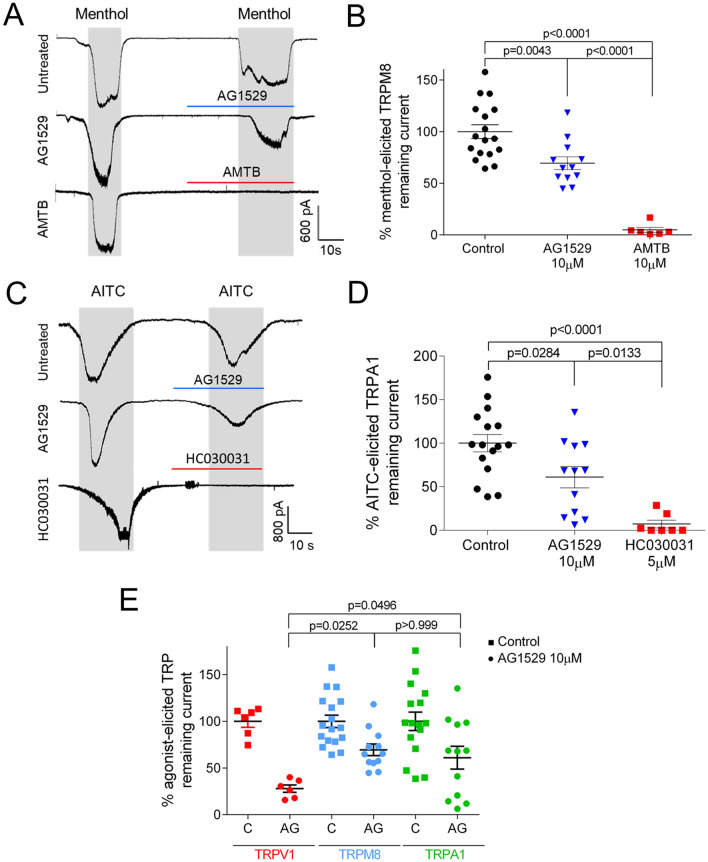
AG1529 marginally inhibits TRPM8 and TRPA1 receptors. (**A**) Representative menthol (100 µM)-elicited hTRPM8 inward currents recorded at a holding potential of − 60 mV, for control cells (Untreated) exposed to two menthol pulses; and, for cells treated with 10 µM AG1529 (AG1529) or 10 µM AMTB (AMTB) for 30 s before the second menthol pulse. (**B**) Percentage of menthol-elicited TRPM8 remaining ionic current with respect to the first menthol pulse. Normalized responses were estimated as (I_Menthol2_/I_Menthol1_) × 100. (**C**) Representative AITC (60 µM)-elicited hTRPA1 inward currents recorded at a holding potential of − 60 mV, for control cells (Untreated) exposed to two AITC pulses; and, for cells treated with 10 µM AG1529 (AG1529) or 5 µM HC030031 (HC030031) for 30 s before the second AITC pulse. (**D**) Percentage of TRPA1 ionic current with respect to the first AITC pulse. Normalized responses were estimated as (I_AITC2_/I_AITC1_) × 100. Grey rectangles denote the duration of the agonist pulses and horizontal lines the duration of the antagonists pulses. All data are expressed as mean ± SEM. **(E**) Comparison of 10 µM AG1529 blockade of hTRPV1, hTRPM8 and hTRPA1. Data were analysed using the One-Way Anova followed by the Bonferroni´s multiple comparison post-hoc test, p-values for statistical differences are indicated. Detail data of the statistical analysis is described in the main text.

ThermoTRP cross-interaction has been reported for modulators of TRPV1, TRPM8 and TRPA1 channels^32,33^. Consequently, we evaluated the effect of AG1529 on recombinantly expressed hTRPM8 and hTRPA1 channels. As exhibited in Fig. 3A, 10 μM AG1529 reduced menthol-evoked ionic currents, although to a lower extend than the reference TRPM8 blocker N-(3-Aminopropyl)-2-[(3-methylphenyl)methoxy]-N-(2-thienylmethyl) benzamide hydrochloride (AMTB) at 10 μM. Quantitation of this inhibitory activity shows that AG1529 blocked TRPM8 channel activity by ≈40% (Fig. 3B), which is a significantly lower efficacy than that of AMTB (F(2,32) = 37.57, p < 0.0001, with p = 0.0043 for AG1529 vs control and p < 0.00001 for AG1529 vs AMBT, Bonferroni´s post-hoc test).

To investigate if AG1529 affected TRPA1-mediated currents, we used allyl isothiocyanate (AITC) as TRPA1 agonist (Fig. 3C). As depicted in Fig. 3C, 10 μM AG1529 inhibited ≈ 40% of AITC-evoked ionic currents, indicating an effect on hTRPA1 channels (Fig. 3D; F(2,32) = 15.67, p < 0.0001; control vs. AG1529, p = 0.0284, Bonferroni´s post-hoc test). In contrast, 5 μM of 1,2,3,6-Tetrahydro-1,3-dimethyl-N-[4-(1-methylethyl) phenyl]-2,6-dioxo-7H-purine-7-acetamide, 2-(1,3-Dimethyl-2,6-dioxo-1,2,3,6-tetrahydro-7H-purin-7-yl)-N-(4-isopropylphenyl)acetamide (HC030031), a reference TRPA1 antagonist, completely abrogated AITC-evoked ionic currents (Fig. 3D; Control vs. HC030031, p < 0.0001, Bonferroni´s post-hoc test). The potency of AG1529 blocking TRPA1 was also significantly lower than that of HC030031 (Fig. 3D; AG1529 vs. HC030031, p = 0.0133, Bonferroni´s post-hoc test).

An statistical comparison of 10 μM AG1529 efficacy blocking the three human thermoTRPs revealed a significantly lower inhibition of TRPM8 and TRPA1 channels as compared with hTRPV1 (Fig. 3E, F(2,27) = 4.434, p = 0.0216; TRPV1 vs. TRPM8, p < 0.0252 and TRPV1 vs. TRPA1, p < 0.0496, Bonferroni´s multiple comparison post-hoc test). Collectively, these data indicate that AG1529 preferentially, but not selectively, interacts on recombinant hTRPV1 channels, exhibiting a milder inhibitory effect on recombinant hTRPM8 and hTRPA1 channels.

### AG1529 interacts with the vanilloid binding site in TRPV1

**Figure 4:**
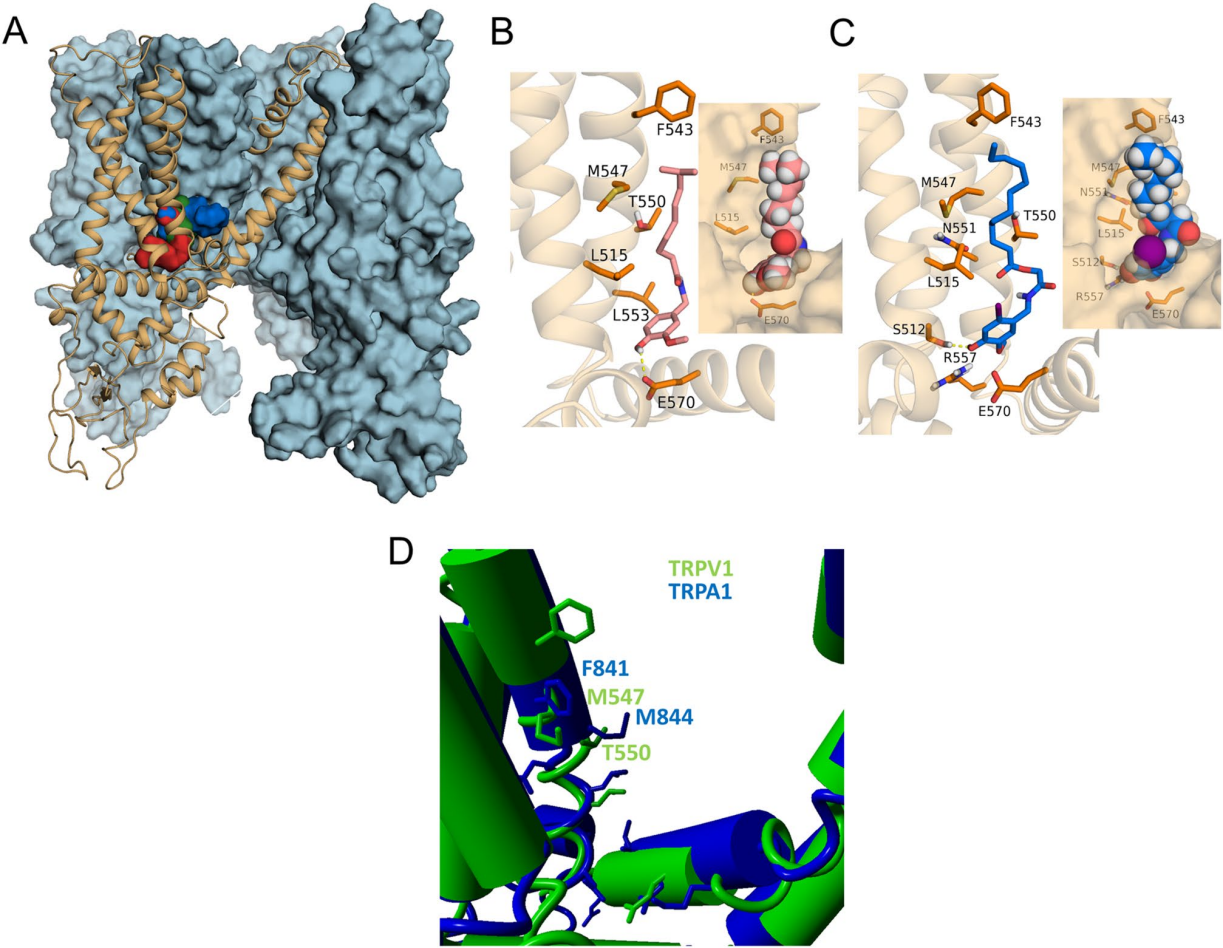
AG1529 binds to the capsaicin binding site in TRPV1. (**A**) Transmembrane view of rat TRPV1 structure with three subunits shown as blue surface and one as orange cartoon. For global blind docking assay capsaicin (red surface), capsazepine (green surface) and AG1529 (blue surface) displayed the same binding pocket which corresponds to the capsaicin binding site compressed between helices S3, S4 and the S4–S5 linker. (**B**) Capsaicin (reddish sticks) orientation in its binding site explored through local docking assay. VDW interactions are observed within capsaicin tail and residues L515 (S3), F543, M547, T550 and L553 (S4). Hydrogen bond is formed between capsaicin head and E570 from the S4-S5 linker. In the inset capsaicin is represented as spheres and the receptor as surface. (**C**) AG1529 (blue sticks) adopted the same orientation as capsaicin and displayed the same VDW interactions but formed a hydrogen bond with S512. In the inset AG1529 is represented as spheres, where the purple one stands for iodine group and receptor is shown as surface. It can be observed the conformation limitation the head of AG1529 experiments. (**D**) Structural view of the capsaicin binding pocket of TRPV1 (green) aligned with TRPA1 (blue). Notice that the M844 side chain of TRPA1 is oriented to the central binding cavity sterically perturbing the accessibility of this site to AG1529. Up to 82% of AG1529 docking trials in TRPA1 failed.

The results on recombinant hTRPV1 suggest that AG1529 may act as a competitive capsaicin antagonist. To further support this tenet, we used molecular modelling to evaluate the putative interaction of AG1529 with the capsaicin binding site in TRPV1 (Fig. 4). We used the TRPV1 atomic structure in its open and closed states and dock AG1529 into the vanilloid binding site to explore the energetics of compound binding and compare it with capsaicin and capsazepine (Fig. 4). The binding energy (mean ± SD) of AG1529 was 3.7 ± 1.3 kcal/mol (n = 116 trials), which was comparable to those of capsaicin 6.4 ± 0.6 kcal/mol (n = 212 trials) or capsazepine 9.3 ± 1.1 kcal/mol (n = 158 trials). No differences in terms of best binding site between open and resting states were observed. Inspection of the interactions reveals that, akin to capsaicin, AG1529 may adopt a tail-up, head-down conformation displaying the aliphatic tail Van der Waals interactions with the same key residues, i.e. L515, F543, M547, T550 and L553 in S3 and S4 transmembrane segments. Hydrogen bonds are formed between the vanillyl head and S512 or E570 residues which grant some specificity to the interaction. A similar docking strategy with TRPA1 showed that the equivalent binding pocket is sterically disturbed due to the central orientation of the M844 side chain (M547 in TRPV1; Fig. 4D), affecting the AG1529 binding affinity. This is in accordance with the moderate potency of AG1529 inhibiting TRPA1 activity. Thus, our modelling approach further suggests that AG1529 is a competitive TRPV1 antagonist that binds to the capsaicin binding site to block channel gating.

### AG1529 blocks capsaicin-evoked neuronal firing

**Figure 5:**
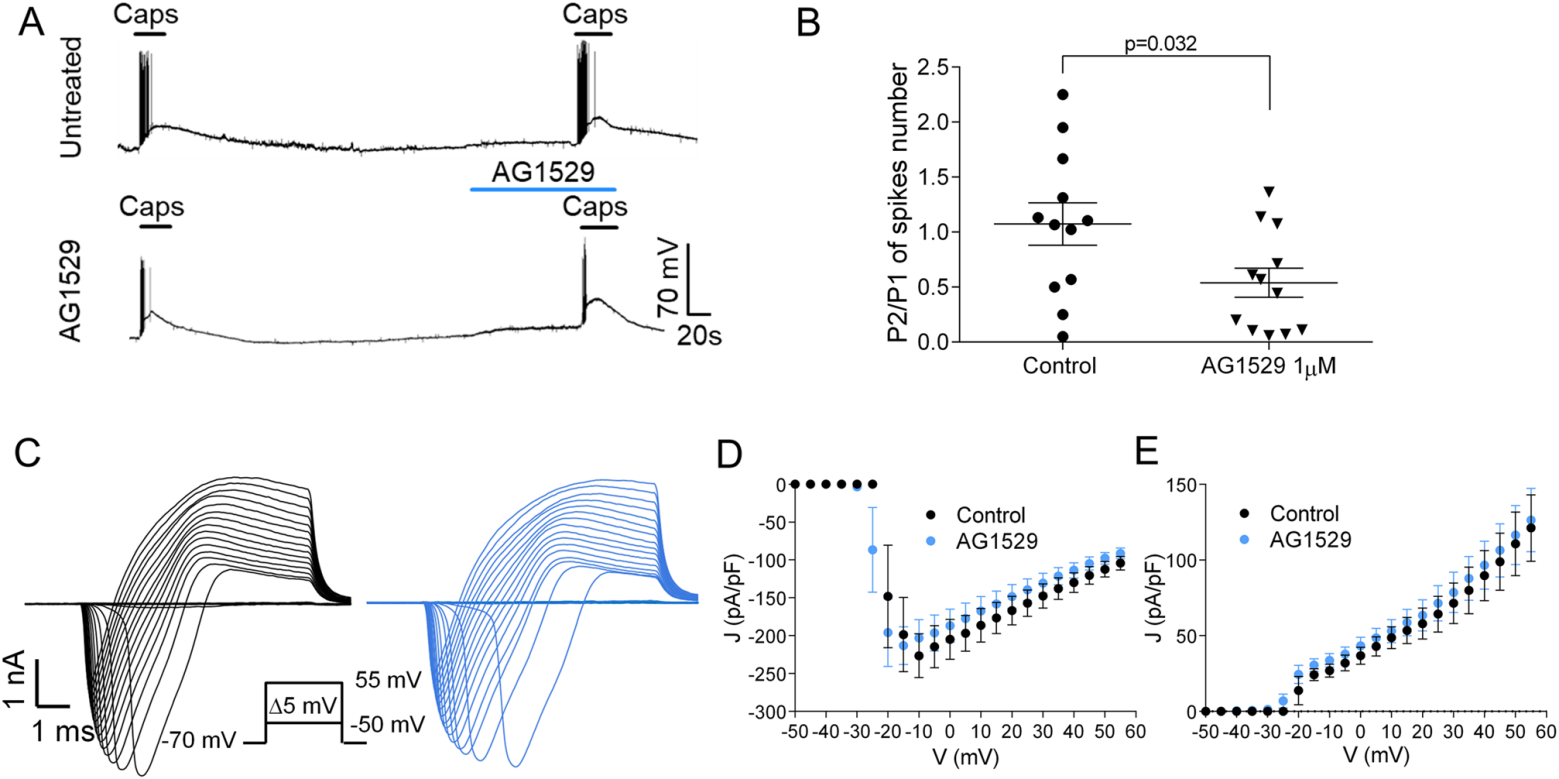
AG1529 affects capsaicin-evoked nociceptor firing. (**A**) Representative recordings of APs firing from DRG neurones elicited by 0.5 μM capsaicin in the absence (Untreated) and presence (AG1529) of 1 µM AG1529. APs were recorded under current clamp mode. Horizontal lines denote the duration of compound pulse. (**B**) Ratio of second (P2) versus first (P1) peak of APs evoked by capsaicin application in the absence (n = 12) and the presence during the P2 of 1 µM AG1529 (n = 12). (**C**) Representative macroscopic ionic currents evoked by a series of depolarizing 5 mV voltage steps of 10 ms, from − 50 mV to 55 mV from a holding potential of − 70 mV, in the absence (black) and the presence (blue) of 1 µM AG1529. (**D**) and (**E**) current density to voltage relationships for the inward and outward currents of panel (**C**), respectively. All data are expressed as mean ± SEM. Data depicted in panel (**B**) was analysed using an unpaired, two-tail Student’s t-test. p-value is indicated.

Next, we investigated the effect of AG1529 on capsaicin-evoked action potentials (AP) in primary cultures of rat DRG sensory neurons. Under current-clamp, small sensory neurons exposed to 0.5 μM capsaicin triggered bursts of APs (Fig. 5A, top trace). The ratio of electrical activity evoked by 2 short capsaicin pulses was variable (Fig. 5B), likely reflecting the phenotypic heterogeneity of DRG sensory neurons. Pre-exposure of small sensory neurons under study to 1 μM AG1529 before the second capsaicin pulse produced a reduction in AP firing (Fig. 5A). Evaluation of the AP frequency ratio between the first (P1) and second (P2) capsaicin pulses reveal a significant 50% reduction in AP firing by 1 μM AG1529 (Fig. 5B; p = 0.032, unpaired, two-tail, t-Student, n = 12).

To ascertain whether this putative effect on neuronal excitability was due to an off-target blockade of Na^+^ and K^+^ voltage-gated channels, we performed voltage-clamp experiments to investigate whether AG1529 affected these ion channels expressed in the sensory neurons. Figure 5C depicts families of putative Na^+^ and K^+^ ionic currents evoked by 5 mV voltage steps from -50 mV to 55 mV in the absence (black) and presence of 10 μM AG1529 (blue). As seen, currents in the absence and presence of the antagonist look alike in terms of magnitude and voltage-sensitivity. Current density-to-voltage relationships for both ionic conductances further corroborated that AG1529 did not affect the voltage-activation properties of Na^+^ and K^+^ conductances (Fig. 5D,E).

### Effect of AG1529 in neuronal electrical activity

Current-clamp measurements obtained with patch-clamp indicate that AG1529 inhibits capsaicin-evoked AP firing in sensory neurons. To further interrogate AG1529 action on the excitability of DRG neurons, we next used multielectrode arrays (MEA). This technology allows recording bursts of action potentials from neuronal populations that are located near to an electrode, but it cannot differentiate the number of neurons or other cells contributing to it. We used MEA chips having 60 electrodes and monitored the electrical activity at each electrode.

**Figure 6:**
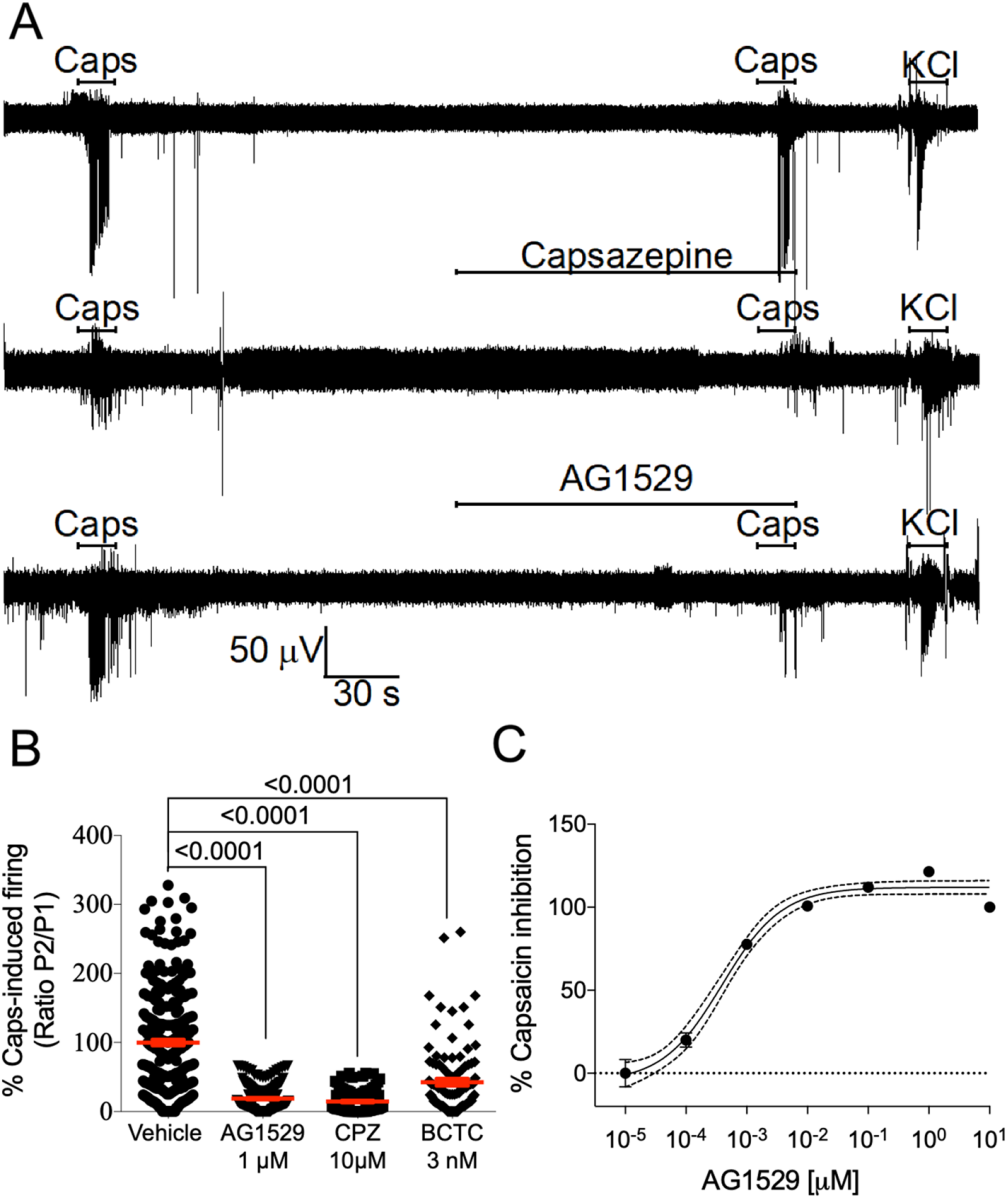
AG1529 attenuates capsaicin-induced nociceptor excitability. (**A**) Representative MEA recordings showing the capsaicin-evoked AP firing for control (top), 10 µM capsazepine (middle) and 1 µM AG1529 (bottom). Capsaicin (0.5 µM) was applied in two sequential pulses of 15 s interspersed with a washing period. Compounds were added 2 min before the second capsaicin pulse. The protocol was terminated with a 15 s-pulse of 40 mM KCl to ensure viability of neuronal cultures. (**B**) Normalized capsaicin-induced firing (P2/P1) in the absence (vehicle) and the presence of the antagonists (1 µM AG1529, 10 µM capsazepine, and 3 nM BCTC). (**C**) Dose–response curve of AG1529 blockade of capsaicin-induced AP firing. Data points were fitted to the Michaelis–Menten isotherm, giving an IC_50_ value of 0.4 nM (95% CI, 0.3 to 0.6 nM) and an n_H_ of 0.88 (95% CI 0.64 to 1.12). The boundary lines denote the interval confidence of the mean values. Data on panel (**B**) were analysed using the One-Way Anova (F(3,699) = 140.7, p < 0.0001) followed by the Bonferroni´s post-hoc test, p-values for statistical difference are indicated. N (independent experiments) = 3, number of electrodes 100–250 per condition).

Figure 6A (top panel) depicts a typical MEA recording from one electrode. The experimental paradigm consisted in applying two 15 s pulses of 0.5 μM, interspersed by a 4.30 min washing step, ending with a 15 s pulse of 40 mM KCl to ensure that the electrical activity near the electrodes was preserved at the end of the protocol. Capsaicin-evoked bursts of neuronal AP firing in ≈70% of the electrodes. Capsaicin-evoked electrical activity was variable as evidenced by the dispersion of the P2/P1 spike frequency ratio (Fig. 6B), likely reflecting the neuronal heterogeneity of the DRG cultures. Vanilloid-induced AP firing was primarily due to TRPV1-mediated neuronal depolarization because capsaicin-evoked neuronal firing of the second pulse was blocked by 10 μM capsazepine or 3 nM BCTC (Fig. 6A, middle panel, and Fig. 6B). Similarly, when 1 μM AG1529 was instilled before the second capsaicin pulse, neuronal firing was significantly reduced (Fig. 6A, lower panel and Fig. 6B; F(3,699) = 140.7, p < 0.0001; p < 0.0001 for treatments vs. control, Bonferroni´s post-hoc test). AG1529 inhibitory activity was dose-dependent, achieving 50% at 0.39 ± 0.08 nM and > 98% at 1 μM (Fig. 6C). The Hill coefficient was n_H_ = 0.9 ± 0.1.

**Figure 7:**
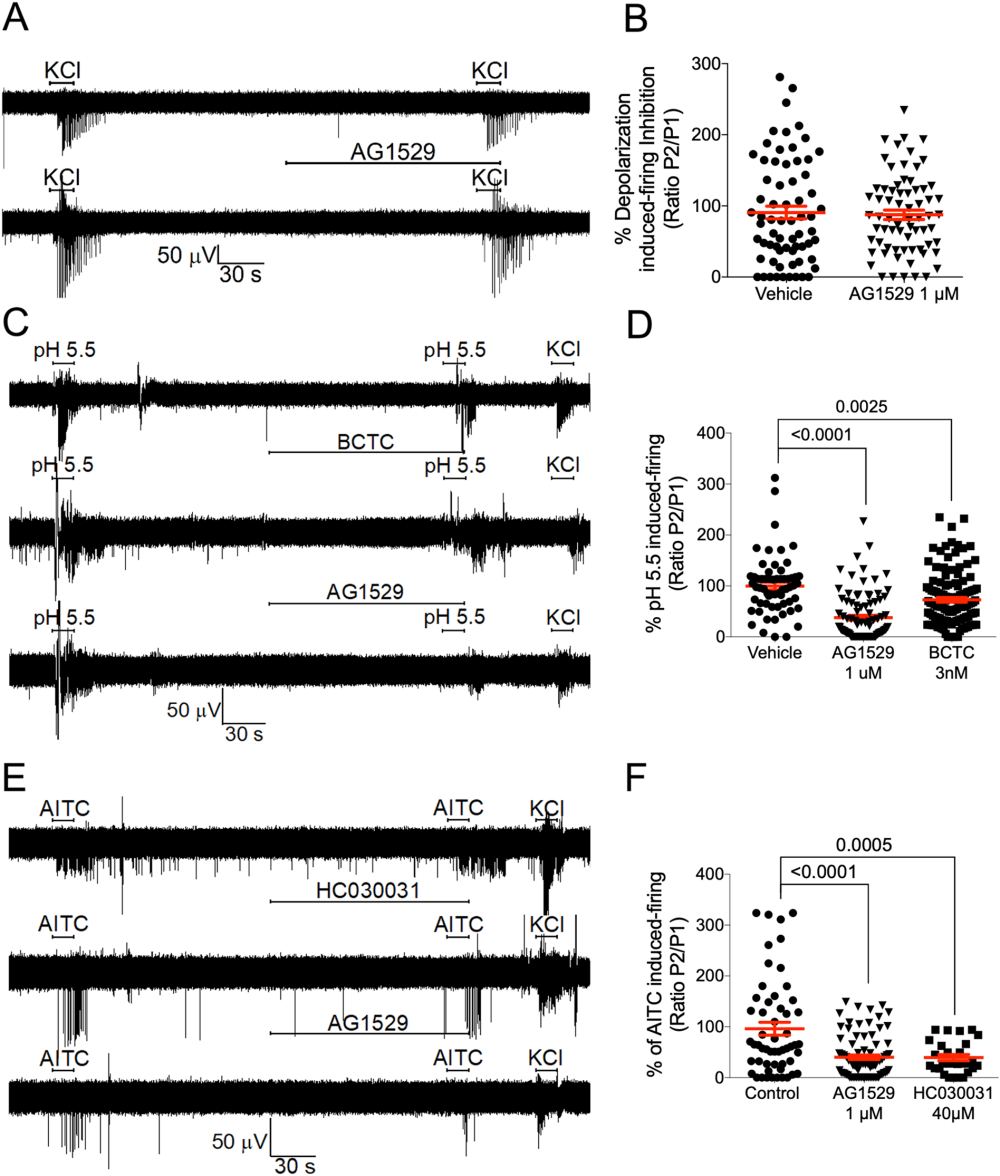
AG1529 effect on voltage, pH and AITC-evoked nociceptor excitability. (**A**) Representative MEA recordings of extracellular K^+^ (40 mM KCl)-induced nociceptor firing of APs in the absence and presence of 1 µM AG1529, applied 2 min before the second extracellular K^+^ pulse. (**B**) Normalized extracellular K^+^-induced neuronal firing of the second pulse in the absence and presence of AG1529. (**C**) Representative MEA recordings of acid pH (5.5)-induced nociceptor firing of APs in the absence and presence of 1 µM AG1529, applied 2 min before the second acidic stimulus. (**D**) Normalized pH-induced neuronal firing of the second pulse in the absence and presence of AG1529. Data were analysed using the One-Way Anova (F(2,275) = 28.15, p < 0.0001). (**E**) Representative MEA recordings of AITC (50 µM)-induced nociceptor firing of APs in the absence and presence of 1 µM AG1529, applied 2 min before the second AITC pulse. (**F**) Normalized AITC-induced neuronal firing of the second pulse in the absence and presence of 1 µM AG1529. Data were analysed using the One-Way Anova F(2,163) = 14.73, p < 0.0001 for panel (**D**) followed by the Bonferroni´s post hoc test, p-values for statistical difference are indicated. N (independent experiments) = 3, number of electrodes = 50–130 per condition.

AG1529 inhibitory activity of capsaicin-evoked AP firing was attained through inhibition of vanilloid-induced TRPV1 activity, as depolarization-evoked firing triggered by increasing the extracellular KCl to 40 mM was not affected by 1 μM AG1529 (Fig. 7A,B). Nonetheless, it should be stated that a marginal blockade of K^+^ evoked firing activity (≈25%) was observed at AG1529 concentrations ≥ 10 μM.

Akin to the in vitro assays with recombinant hTRPV1, AG1529 also attenuated pH-induced AP firing of rat DRG neurons, although to a much weaker extent compared to the effect on capsaicin-evoked firing (Fig. 7C,D; F(2,275) = 28.15, p < 0.0001; p < 0.0001, AG1529 vs. control and p = 0.0025, BCTC vs. control, Bonferroni´s post-hoc test). It should be considered that pH-induced neuronal excitability may be contributed by both TRPV1 and ASIC channels. Thus, the variable activity recorded in the electrodes that remain insensitive to AG1529 and BCTC (Fig. 7D) may correspond to that triggered by ASIC channels.

We also investigated whether AG1529 affected nociceptor firing evoked by AITC, presumably through activation of TRPA1 channels (Fig. 7E). Indeed, AITC increased AP firing and this effect was sensitive to blockade by HC30031 (Fig. 7E,F). We observed that AG1529 at 1 μM inhibited ≈65% of the AITC-induced neuronal activity (Fig. 7E,F; F(2,163) = 14.73, p < 0.0001; p < 0.0001, AG1529 vs control and p = 0.0005, HC030031 vs control, Bonferroni´s post-hoc test). This inhibitory activity was similar to that exerted by capsazepine (67 ± 5%) and BCTC (58 ± 17%) on AITC evoked nociceptor firing, suggesting a potential cross-talk between TRPV1 and TRPA1 channels that are co-expressed in the same subpopulation of nociceptors, or to the proposed presence of heteromeric channels^34^. A dose response for AG1529 blockade of AITC firing revealed an IC_50_ of 0.3 ± 0.2 μM that is 1,000-fold higher than that estimated for capsaicin-evoked nociceptor firing using this methodology. The Hill coefficient n_H_ was 0.8 ± 0.4. Together, these data indicate that AG1529 is a potent inhibitor of vanilloid-induced, TRPV1-mediated nociceptor excitability, and modestly affects that evoked by TRPA1 activation.

### AG1529 attenuates histamine- and inflammation-induced sensitization of capsaicin-activated sensory neuron firing

**Figure 8:**
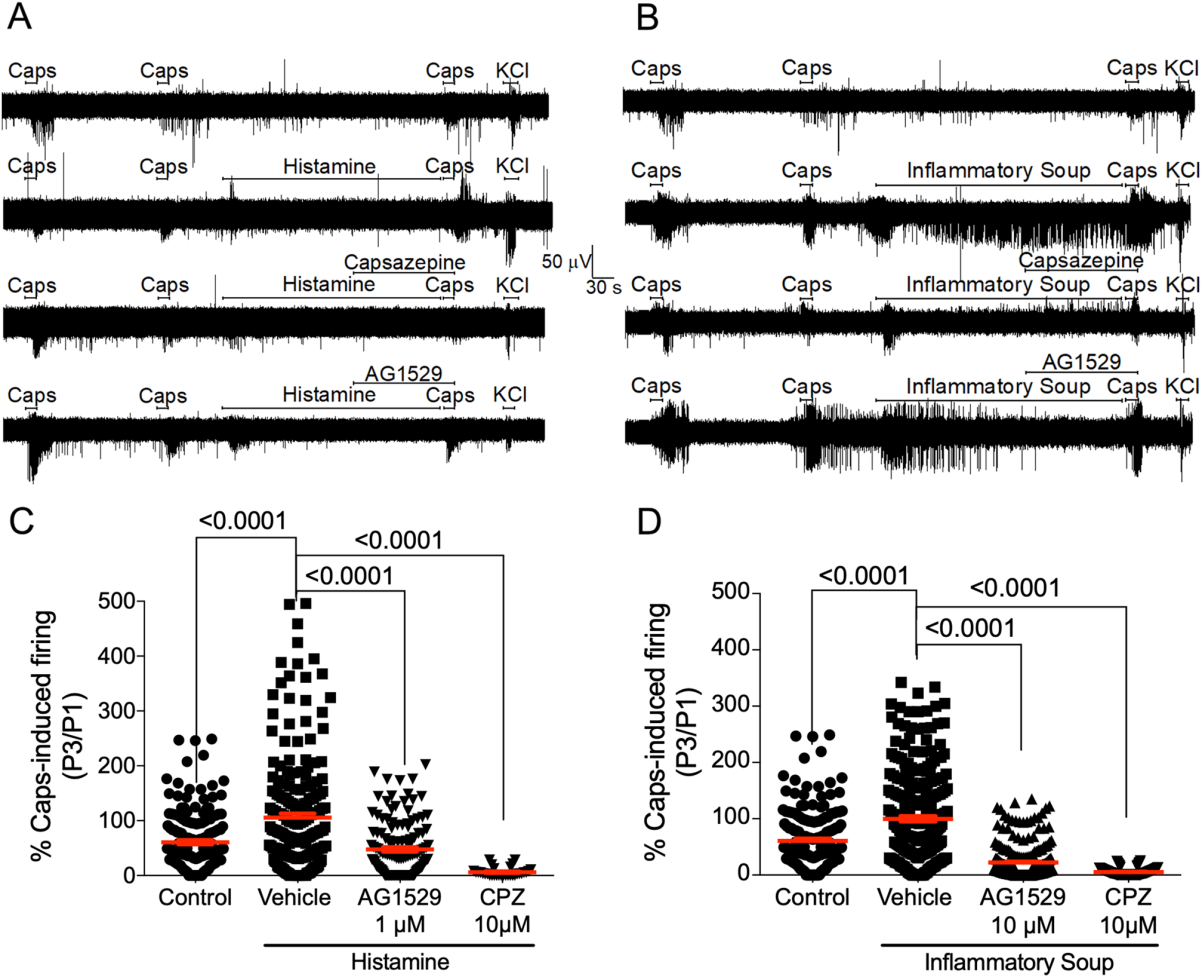
AG1529 reduces algesically-sensitized capsaicin-induced nociceptor excitability. (**A**) Representative MEA recordings of capsaicin-evoked APs and its sensitization by instillation of 50 µM histamine 2 min before the third capsaicin pulse, along with the effect of 10 µM capsazepine or 1 µM AG1529 added 2 min before the third capsaicin pulse (P3). (**B**) Similar protocol that in (**A**) but sensitization was triggered by an inflammatory soup containing 50 µM histamine, 1 µM bradykinin and 10 µM ATP. (**C**,**D**) Normalized capsaicin-induced firing of the third pulse (P3) with respect to the first one (P1) for the different treatments. Data are expressed as mean ± SEM, and analysed using the One-Way Anova (F(3,553) = 28.28, p < 0.0001 for panel (**C**); and F(3,794) = 144.5, p < 0.0001 for panel **D**) followed by the Bonferroni´s post-hoc test, p-values for statistical difference are indicated. N (independent experiments) = 3, nº of electrodes = 50–200). Data of vehicle without histamine or inflammatory soup were obtained in independent experiment, N = 3 and number of electrodes 120.

We next investigated whether AG1529 could attenuate the potentiation of TRPV1 activity by histamine and an inflammatory soup. For this task, we desensitized TRPV1 with two pulses of 0.5 μM capsaicin (P1, P2) and evaluated the effect of the pro-algesic agents on neuronal firing activity evoked by a third vanilloid pulse (P3) (Fig. 8A,B). Histamine and the inflammatory soup were instilled between P2 and P3 for 5 min (Fig. 8A,B). As illustrated in Fig. 8A,B, repeated application of capsaicin-evoked neuronal firing produced some desensitization. Exposure of DRG neurons on MEA chips to 50 μM histamine for 5 min between P2 and P3 capsaicin pulses overcome capsaicin-induced desensitization and produced an increment of the vanilloid-evoked nociceptor firing, consistent with a sensitizing action by histamine (Fig. 8A,C). Histamine-induced potentiation of capsaicin firing was inhibited by 10 μM capsazepine perfused 2 min before P3 (Fig. 8A,C). These results indicate that histamine signaling was primarily mediated by TRPV1 channels. Notably, AG1529 was also very effective blocking histamine-induced sensitization of TRPV1 in DRG cultures (Fig. 8A). At 1 μM, AG1529 reduced by > 60% the histamine sensitized capsaicin-induced AP firing (Fig. 8C, F(3,553) = 28.28, p < 0.0001; p < 0.0001 treatments vs. vehicle, Bonferroni´s post-hoc test).

We also tested the activity of compound AG1529 on capsaicin responses sensitized by an inflammatory soup containing 50 μM histamine, 1 μM bradykinin and 10 μM ATP. As expected, instillation of this inflammatory soup for 5 min between P2 and P3 capsaicin pulses abrogated the vanilloid-induced desensitization and promoted a notable potentiation of capsaicin evoked neuronal firing (Fig. 8B,D). Inflammatory potentiation of capsaicin responses was blocked by capsazepine (Fig. 8B,D). Akin to capsazepine, 10 μM AG1529 administered 2 min before and during the third capsaicin pulse significantly reduced the vanilloid sensitized AP firing (Fig. 8D; F(3,794) = 144.5; p < 0.0001; p < 0.0001, treatments vs. vehicle, Bonferroni´s post-hoc test), thus attenuating the inflammatory potentiation of TRPV1 in DRG neurons. Collectively, these results substantiate an anti-pruritic and anti-inflammatory activity of AG1529 by modulating TRPV1 activity in sensory neurons.

### Topically applied AG1529 reduces histamine-induced pruritus

**Figure 9:**
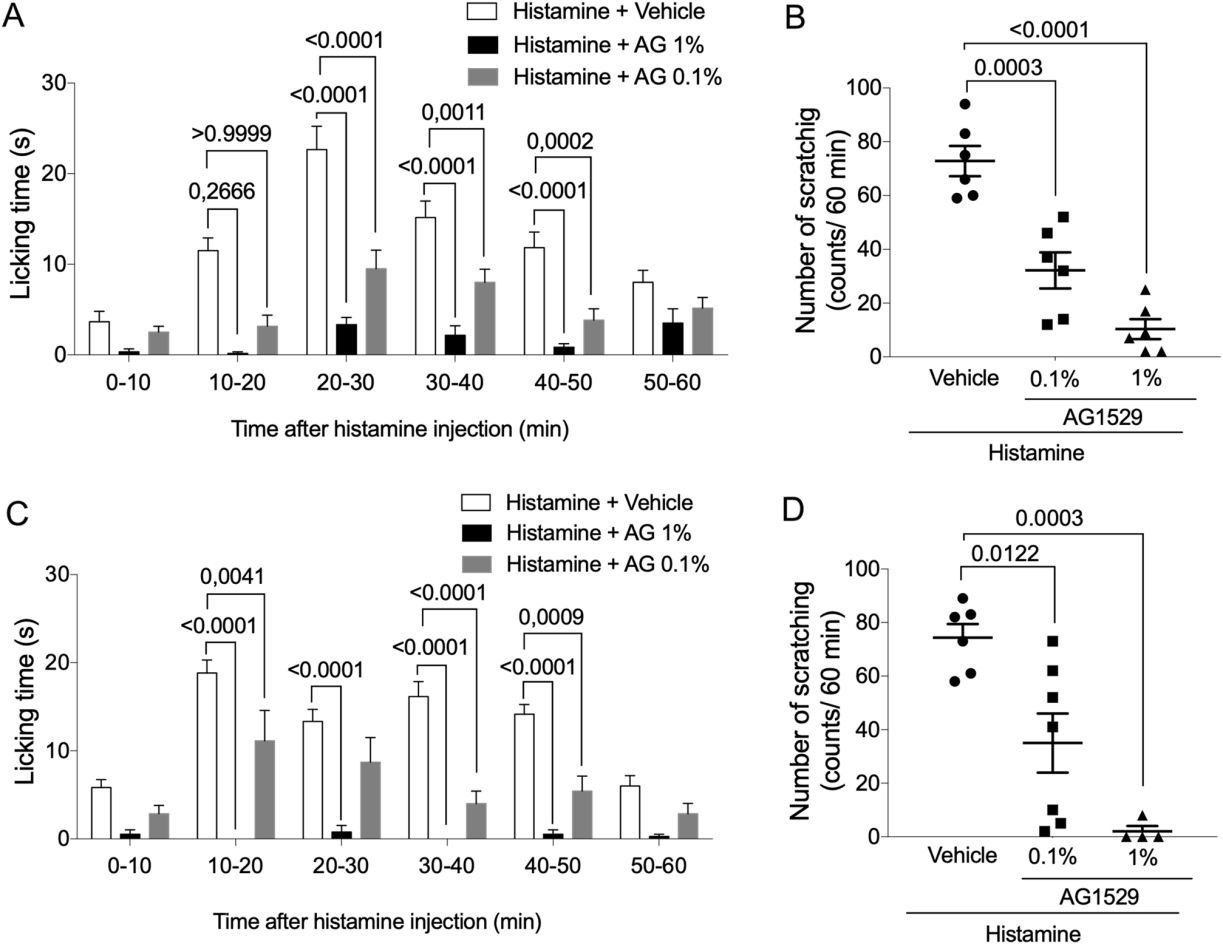
Topical application of AG1529 inhibits histamine-evoked pruritus. Effect of AG1529 (0.1 and 1%) dissolved in acetone (**A**,**B**) or formulated in a dermal ointment (**C**,**D**) on the scratching behavior of rats locally injected with histamine in the back of the neck. Animals were injected with 100 µl of a solution at 1 mg/ml of histamine. AG1529 in acetone was wiped in 1 cm^2^ area, 30 min before the histamine instillation. Ointment containing AG1529 was applied topically twice/day for 3 days with a spatula in a 1 cm^2^ shaved area in the back of the neck. Data are expressed as mean ± SEM, n = 6 animals per group. Data were analysed using the One-way Anova followed by the Bonferroni´s post-hoc test, p-values for statistical difference are indicated. Detail data of the statistical analysis is described in the main text.

Our in vitro findings, using histamine and an inflammatory soup containing the pruritogenic agent (Fig. 8), alongside with the previously reported in vivo antinociceptive activity of AG1529 when administered systemically (i.v.) or locally (intraplantar)^31^, prompted us to question whether dermal application of AG1529 could have therapeutic value. For this experiment, we designed two formulations that are compatible with the low water solubility of AG1529. Firstly, we dissolved the compound in acetone and directly wiped the skin of the animals before local administration of histamine. To monitor the licking activity as a function of time, the 60 min observation period was binned in 10 min intervals (Fig. 9A). As depicted, pruritic activity of histamine was very evident from 10 to 50 min after administration. AG1529 at 0.1% and 1% significantly attenuated the licking time during the entire time interval. Furthermore, AG1529 wiped on the skin reduced the total number of scratches provoked by histamine in a dose dependent manner (Fig. 9B; F(2,15) = 33.35, p < 0.0001; p = 0.0003, 0.1% vs vehicle; p < 0.0001, 1% vs vehicle; and, p = 0.0395, 0.1% vs 1%, Bonferroni´s post-hoc test), indicating that dermal application of the product exerted an anti-pruritic effect.

To further substantiate the translational clinical potential of AG1529, we formulated the compound in an anhydrous ointment and tested its therapeutic efficacy in the rat model of acute histaminergic pruritus. To ensure the skin permeation of the compound, we rubbed the AG1529 ointment previously to the administration of histamine. Dermal application of the AG1529 ointment did not produce any nocifensive response of the rats, substantiating that AG1529 does not produce the burning sensation characteristic of capsaicin. As depicted in Fig. 9C,D, animals pre-treated with the ointment base (vehicle) exhibited a high number of scratches provoked by the pruritogenic agent. In marked contrast, application of the ointment containing AG1529 significantly attenuated the scratching behavior (Fig. 9C,D), with full abrogation at 1% of AG1529 (Fig. 9D; F(2,14) = 15.28; p = 0.0003; p < 0.0122, 0.1% vs vehicle; p = 0.0003, 1% vs vehicle, Bonferroni´s post-hoc test). Therefore, these results signal to dermal formulations containing AG1529 as potential therapeutics for treating pruritus.

## Discussion

TRPV1 is a therapeutic target for modulating nociceptive signaling, including pain and itch. A major effort has been devoted to design therapeutically useful antagonists for the treatment of chronic pain and pruritus. However, most of the highly potent antagonists developed have failed in clinical trials because of unwanted side-effects, primarily hyperthermia^35^. In contrast, the desensitizing action of capsaicin is widely used in the clinic for the treatment of chronic pain and pruritus, but with poor patient adherence due to the burning sensation and the potential carcinogenesis because of its poor skin elimination^36^. Thus, there is interest in developing capsaicin-like molecules devoid of the flaming sensation while preserving the therapeutic action. Previously, we reported the design of soft drugs based on the capsaicinoid scaffold as TRPV1 antagonists that neither show the burning nocifensive response in animals, nor alter the body temperature^31^. Soft drugs are a kind of compounds that are metabolically deactivated by dermal and plasma esterases to inert metabolites, preventing their tissue accumulation and favoring their body elimination^31^. Here, we characterized the effect of soft compound AG1529 on hTRPV1 activity to evaluate its potential clinical translation. In addition, we also assessed the possible therapeutic use of AG1529 as a dermal anti-pruritic drug. We show that AG1529 is a hTRPV1 antagonist that reversibly blocks the channel with micromolar potency. This activity was modestly receptor selective as AG1529 also inhibited hTRPA1 and hTRPM8 receptors, although with lower potency than hTRPV1. Noteworthy, we found that AG1529 preferentially inhibited capsaicin-evoked TRPV1 gating, marginally affected pH-mediated channel activation, and it did not alter voltage- and heat-induced gating. Note that the modest inhibition of pH-evoked TRPV1 responses, and lack of effect on TRPV1 thermal activation may underlie the non-hyperthermic in vivo activity of AG1529^31^. Furthermore, the lack of effect on heat-evoked gating suggests that AG1529 may not alter cutaneous temperature sensitivity, which is pivotal for the topical therapeutic application of the compound. Thus, AG1529 is a hTRPV1 antagonist exhibiting in vitro functional properties with a potential clinical translation.

Our functional in vitro data support the tenet that AG1529 acts as a capsaicin competitive antagonist. Firstly, it displaced the capsaicin EC_50_ towards a higher value; and, secondly, it attenuated capsaicin-induced desensitization of hTRPV1. A molecular model further substantiated this mechanism of action showing that AG1529 binds with high theoretical energy to the capsaicin binding site in the receptor. Considering this molecular model, it is tempting to use it for hypothesizing a mechanism for the inhibitory activity of AG1529. It has been proposed that capsaicin induces TRPV1 gate opening by pulling of E570 through hydrogen bond formation with the residue which causes the final motion of S6 leading to gate opening^37^. The presence of an iodine group in the vanillyl head of AG1529 appears responsible for the competitive antagonism of AG1529. Indeed, the interaction of the compound with residue E570 seems compromised by the bulky 2.16 Å iodine group that limits the capacity of AG1529 to adopt a head orientation akin to capsaicin (Fig. 4). Note that the orientation driven by the Iodine in the vanilloid group probably induces an electrostatic repulsion with Glu-570 in the S4-S5 loop thus relocating the vanilloid group close to S3. This configuration, along with the lack of interaction with the S4-S5 loop seems to lock the channel in the closed configuration. In support of this hypothesis, elimination of the Iodine from the vanillyl group brings closer the vanilloid group to Glu-570 in the S4-S5 loop, consistent with the agonist activity displayed by this capsaicinoid molecule^31^. Ongoing structure–function studies in our laboratory are testing this model for the interaction of AG1529 with TRPV1.

For therapeutic use is essential that TRPV1 antagonists are capable of modulating nociceptor excitability, particularly under pro-algesic/inflammatory conditions. Our studies reveal that AG1529 effectively attenuates capsaicin-induced nociceptor firing in single sensory neurons and neuronal networks. It is intriguing that the AG1529 potency blocking capsaicin-evoked electrical activity in neuronal networks seeded in MEA chips was notably higher than that inhibiting hTRPV1-mediated ionic currents in recombinant HEK293 cells. This discrepancy does not appear related a different sensitivity of the human and rat orthologs as they are blocked with similar IC_50_ values (hTRPV1, 1.2 ± 0.1 μM (n = 16) and rTRPV1, 0.9 ± 0.2 μM (n = 13), determined by Ca^2+^ microfluorography). Notably, a similar increase in blocking potency of AG1529 was obtained on AITC-evoked electrical activity in neuronal networks. We do not have a solid explanation for the magnitude of these differences in potency recorded by both techniques. One possibility is that it could be contributed by the different cellular environments (recombinant vs neuronal), and/or that the MEA mean electrical activity reflects the electrical activity evoked by the heterogeneous diversity of sensory neurons near an electrode. In contrast, patch-clamp measures individual cells in homogeneous cell populations. Nonetheless, we cannot discard that the higher blocking potency of AG1529 in primary cultures of nociceptors may be also due to off-target actions of the compound that affect the firing of action potentials. In this regard, we observed that 10 μM AG1529 reduced the electrical activity evoked by 40 mM KCl (data not shown). Thus, additional studies are needed to investigate the actions of AG1529 in neuronal excitability to unveil potential off-targets effects in its neuronal activity. It should be noted that apart from the quantitative differences, data obtained in recombinant cells are fairly reproduced in the DRG cultures.

We also found that AG1529 display an inhibitory activity on TRPM8 and TRPA1 channels, although with lower potency. Ligand cross-recognition between these receptors has been reported, and it appears that it could be due to conservation of transmembrane binding pockets capable to accommodate these molecules^32,33,38^.Our results with AG1529 further support this tenet. It is also interesting the activity on TRPA1 as the presence of heteromeric TRPV1-TRPA1 receptors has been reported in sensory neurons^34,39^. It is worth noticing that this inhibitory action on TRPA1 activity may underlie the attenuation of histaminergic and non-histaminergic pruritus exhibited by AG1529, as pruritus is a very complex, multifactorial sensory process involving the contribution of both TRPV1 and TRPA1 channels^40^. Accordingly, our findings suggest that compound cross-reactivity among thermoTRPs may have a therapeutic advantage in multifactorial disorders such as pain and pruritus compared to highly selective antagonists acting on one target.

A salient contribution of our findings is that AG1529 potently reduced the potentiation of capsaicin-evoked neuronal firing provoked by a pro-inflammatory soup containing bradykinin, ATP and histamine, as well as by histamine alone. This result substantiates a therapeutic potential of AG1529 for attenuating that neurogenic inflammation underlying cutaneous diseases, as well as algesic and pruritogenic conditions that increase sensory neuron excitability. Thus, we evaluated the efficacy of a dermal AG1529 application, considering that soft drugs are designed to be metabolically deactivated in plasma upon exerting their therapeutic action^31^. We found that topically applied AG1529, formulated in a pharmaceutical ointment, remarkably reduced acute histaminergic itch at therapeutic doses (< 1%). These findings complement previous results showing that intraplantar administration of AG1529 diminished both histaminergic and non-histaminergic pruritus^31^. Nonetheless, the full therapeutic potential of AG1529 needs to be address in chronic models of pruritus exhibiting a multifactorial etiology that leads to excitation of peripheral nociceptor terminals encoding the pruritogenic signal.

In conclusion, our in vitro preclinical results imply that AG1529, akin to PAC14028^41,42^, may be a valuable drug candidate for the treatment of the itching symptoms of dermatological diseases such as dermatitis or psoriasis, along with cutaneous inflammation. AG1529 is a TRPV1 antagonist that does not produce hyperthermia and its deactivation by cutaneous esterases prevents its long accumulation in the skin thus reducing any adverse long-term effect^31^. Furthermore, this capsaicinoid should not alter cutaneous thermal sensitivity as it does not affect heat-evoked TRPV1 gating. Noteworthy, dermal application of the pharmaceutical ointment did not produce a nocifensive or irritating response in the animals, nor any apparent erythema-like cutaneous effect on the shaved back neck skin upon repetitive applications for 3 days suggesting a suitable cutaneous safety for this capsaicinoid. As a result, AG1529 is currently in regulated pre-clinical safety studies, anticipating its potential clinical development for topical treatment of psoriatic pruritus.

## Methods

### Animal experimentation

All procedures were approved by the Institutional Animal and Ethical Committee of the University Miguel Hernández de Elche, in accordance with the guidelines of the Economic European Community, the National Institutes of Health, and the Committee for Research and Ethical Issues of the International Association for the Study of Pain. Animals were kept in a controlled environment (21–23 °C, 12 h light/dark cycle), and had food and water available ad libitum. Neonatal Wistar rats were purchased from UMH in house bred stock (originally from Harlan Laboratories). Adult Wistar rats (100–125 g) were purchased from Harlan Laboratories, Netherlands.

### Primary cultures of DRG nociceptors

Neonatal dorsal root ganglia (DRGs) from Wistar rats (3–5 days-old) were isolated and digested with 0.25% (w/v) collagenase (type IA) in DMEM GlutaMax with 1% (v/v) penicillin/streptomycin (P/S) solution for 1 h (37 °C, 5% CO_2_, ThermoScientific incubator) as previously described^43,44^. After digestion, DRGs were mechanically dissociated. Single cell suspension was passed through a 100 μm cell strainer and washed with DMEM GlutaMax with 10% (v/v) fetal bovine serum (FBS) and 1% (v/v) P/S. Cells were seeded in a drop on microelectrode array chambers previously coated with poly-l-lysine (8.3 μg/mL) and laminin (5 μg/mL). After 1 h, medium was replaced with DMEM GlutaMax, 10% (v/v) FBS and 1% (v/v) P/S, supplemented with mouse 2.5S NGF 50 ng/mL and 1.25 μg/mL cytosine arabinoside (37 °C, 5% CO_2_). All experiments were performed after 48 h cell seeding. All cell culture procedures were performed in a laminar flow cabinet (Model Telstar AV-100).

### Microelectrode array measurements

Extracellular recordings were performed using multiple electrode planar arrays of 60-electrode thin MEA chips, with 30 μm diameter electrodes and 200 μm inter-electrode spacing with an integrated reference electrode (Multichannel Systems GmbH)^44^. The electrical activity of primary sensory neurons was recorded by the MEA1060 System (Multi Channel Systems GmbH1) and MC_Rack software version 4.3.0. Measurement of neuronal firing activity was performed by two different protocols. In the first one, two short 15 s-applications (defined as P1 and P2, respectively) of the stimulus (Capsaicin, AITC, KCl or acidic pH) using continuous perfusion system (2 mL/min) were applied. Between each stimulus cells were washed with external solution for 4 min and 30 s. Treated cells were perfused with AG1529 (obtained from AntalGenics), capsazepine, BCTC, (Sigma Aldrich), or HC030031 (Sigma Aldrich), 2 min before and together with P2. In the second one, as a TRPV1-sensitization protocol, neuronal firing was evoked by three short 15 s applications (defined as P1, P2 and P3, respectively) of 0.5 μM capsaicin using continuous perfusion system. Between P1 and P2, cells were washed with external solution for 2 min and 30 s. Between P2 and P3, after washing for 1 min and 30 s with external solution, cells were treated with 50 µM histamine or an inflammatory soup (50 µM Histamine, 1 µM bradykinin, 10 µM ATP) for 5 min. At the end of each protocol 40 mM KCl was applied to confirm appropriate cell culture excitability and viability. All measurements were performed at ~ 34.5 °C (Multichannel Systems Temperature Controller).

### Microelectrode array analysis

Data were analyzed using MC_RACK spike sorter with a sample rate of 25 kHz and Butterworth high-pass 2nd order filter applied with 200 Hz cutoff. An evoked spike was defined when the amplitude of the neuronal electrical activity was established by automatic threshold estimation at − 5.0 µV Std. Dev. Spiking activity was measured in a temporal interval of 60 s, starting right after instillation of the activating stimuli. Electrodes not displaying electrical activity in the first capsaicin pulse were discarded. The recorded signals were then processed to extract mean spike frequency for each pulse (P1–P3). Then, the ratio P2/P1 or P3/P1 (as indicated) of mean spike frequency was calculated and normalized to vehicle for comparing the different conditions used.

### Patch-clamp recordings from recombinant cells

Whole-cell patch-clamp recordings were made on HEK 293 LTV cell line cultured in DMEM GlutaMAX supplemented with 10% (v/v) fetal bovine serum and 1% penicillin/streptomycin solution, transiently co-transfected with YFP and hTRPV1 or hTRPM8 or hTRPA1 encoding plasmids using Lipofectamine 3000 (Invitrogen) following manufacturer instructions. Transfected cells were registered 2 days after transfection, seeded on 12 mm Ø glass coverslips treated with poly-L-lysine solution (Sigma Aldrich). Intracellular pipette solution contained in mM: 150 NaCl, 3 MgCl_2_, 5 EGTA and 10 HEPES, pH 7.2 with CsOH. Extracellular physiological solution contained (in mM): 150 NaCl, 6 CsCl, 1 MgCl_2_, 1.5 CaCl_2_, 10 glucose and 10 HEPES, pH 7.4 with NaOH. For extracellular acidic solution 10 mM HEPES was substituted with 10 mM MES remaining the rest of components equal to the physiological solution and adjusting to pH 5.5. All measurements were performed at room temperature except heat-evoked TRPV1 activity recordings. Whole-cell experiments were conducted using an EPC-10 amplifier (HEKA Electronik) with Patchmaster software. Patch pipettes, prepared from thin-wall borosilicate capillary glass tubing were pulled with a horizontal flaming/brown Micropipette puller Model P-97 from Sutter Instrument. to a final resistance of 2–8 MΩ when filled with internal solution. Recordings were acquired at 10 kHz and low-pass filtered at 3 kHz. Recordings with leak currents > 200pA or series resistance > 20MΩ were discarded.

In voltage-clamp recordings, cells were maintained at a constant potential and application of distinct modulators was performed. Total currents were always normalized to the first current peak evoked by a stimulus. AG1529 effect on TRPV1 voltage dependence was studied in a voltage step protocol from − 120 mV to 120 mV using 100 ms steps of 20 mV from a holding potential at 0 mV. Leak currents were not subtracted. Conductance was calculated with equation:

$$G=\frac{J}{(V-{V}_{r})}$$

where G is channel conductance, J is the current density (nA/pF), V_r_ (mV) is the reversal potential and V (mV) is the applied voltage.

Heat-elicited TRPV1 currents were evoked by increasing of external bath temperature to 43 °C with a heatable perfusion cannula PH01 programmed with the temperature controller TC02 from Multi Channel Systems MCS GmbH. Ionic currents were recorded with a voltage ramp protocol from − 120 mV to 120 mV in 300 ms. The protocol to evaluate TRPV1 heat-evoked gating inhibition by AG1529 consisted in: (i) record the ionic activity of the channel at 37 °C using the voltage ramp protocol (basal activity); (ii) increase the external bath temperature up to 43 °C and record the channel activity using the voltage ramp protocol (P1); (iii) decrease the temperature until 37 °C and perfuse AG1529 (10 µM), capsazepine (10 µM) or BCTC (1 nM) for 30 s; and, (iv) rise the temperature to 43 °C; and record the ionic currents with the voltage ramp protocol (P2). Basal current (37 °C) was subtracted from the current evoked at 43 °C. The mean P2/P1 ratio for control was used for normalizing the ionic current at 120 mV for all treatments.

To monitor AG1529 (10 µM) effect on pH-evoked TRPV1 currents, ASIC channels activated by acidic pH present in HEK 293 LTV cell line were blocked with 50 µM Amiloride^45^. Currents were evoked at − 60 mV with a pulse of buffer at pH 5.5. The activity of AG1529 was compared to that of capsazepine (10 µM) and BCTC (1 nM).

For capsaicin dose–response relationships, responses were normalized with respect to that evoked in the absence of channel blockers. Capsaicin dose–response curves were fitted to the Michaelis–Menten Isotherm:

$$\frac{I}{{{I_{\max }}}} = \frac{1}{{{{\left( {\frac{1 + [capsaicin]}{{E{C_{50}}}}} \right)}^{n_H}}}}$$

where, EC50 denotes the concentration of capsaicin needed to activate half of the maximal response, and n_H_ denotes the Hill coefficient, which is an estimate of the number of vanilloid binding sites. Dose–response curves for blockade activity were fitted to the Michaelis–Menten Isotherm:

$$\frac{I}{{{I_{\max }}}} = \frac{1}{{{{\left( {\frac{1 + [blocker ]}{{I{C_{50}}}}} \right)}^{n_H}}}}$$

where IC_50_ denotes the concentration of channel blocker that inhibits half of the response obtained in its absence (I_max_). Experimental data were fitted to the Hill equation with a nonlinear least-square regression algorithm with the Prism7 software package.

### Patch-clamp recordings from DRG nociceptors

Whole-cell patch-clamp recordings from sensory DRG neurons from neonatal Wistar rats were carried out 2 days after seeding on 12 mm Ø glass coverslips treated with poly-L-lysine solution and Laminin (Sigma Aldrich). Intracellular pipette solution contained (in mM): 4 NaCl, 126 K gluconate, 0.02 CaCl_2_, 1 MgSO_4_, 5 HEPES, 15 glucose, 3 ATP, 0.1 GTP and 5 EGTA, pH 7.2 with KOH. Extracellular solution contained (in mM): 140 NaCl, 4 KCl, 2 CaCl_2_, 2 MgCl_2_, 10 HEPES, 5 glucose and 20 mannitol, pH 7.4 with NaOH.

Current-clamp recordings of APs generated by the application of two pulses of 500 nM capsaicin in a time interval of 5 min and a 1 min perfusion of 1 µM AG1529 before the second capsaicin pulse, during the injection of a constant depolarizing current that maintained the membrane potential at − 70 mV were used to calculate P2/P1 ratio of AP generation. Data was acquired at a sampling rate of 100 μs and low-pass filtered at 3 kHZ. Cell that did not responded to the first pulse of capsaicin were discarded.

Voltage-gated DRG neuron currents were evoked by stepping from − 50 to 55 mV for 10 ms with 5 mV increments with 2 s interpulse intervals. Inward and outward current densities (nA/pF) were obtained by dividing the peak inward and outward current, respectively, by the cell capacitance. Neurons were voltage-clamped at − 70 mV. AG1529 (1 µM) was perfused during 2 min before recording the step protocol.

### In vivo model of pruritus

AG1529 (0.1 and 1% w/v) dissolved in acetone was applied topically (100 µL) with a syringe 30 min before histamine injection in a 1 cm^2^ area in the back of the neck. Control animals were treated with 100 µL acetone. AG1529 ointment formulation (0.1 and 1% w/w) or placebo cream (8% glyceryl behenate, 8% glyceryl stearate, 4% hydrogenated castor oil 5% diethylene glycol monoethyl ether, 0.1% alpha-tocopherol, 75% caprylic/capric triglyceride) was applied topically twice/day for 3 days with a spatula in a 1 cm^2^ shaved area in the back of the neck. Control animals were treated with placebo cream. Histamine solution (1 mg/ml in saline) was injected subcutaneously in a volume of 100 µL into the shaved area, 30 min after application of AG1529 dissolved in acetone or on day 4 after first AG1529 cream administration. Scratching behavior was observed during 60 min after histamine injection. The number of scratching bouts (counts of scratching/60 min), defined as the action of scratching the affected area, was measured from the start of the first scratching movement.

### Virtual docking assays

Rat TRPV1 crystal structure in open (3.3 Å resolution, pdb code: 5irz) and closed (3.3 Å resolution, pdb code: 3j5p) states were obtained, human TRPA1 (2.9 A resolution, pdb code: 6PQO) from the Research Collaboratory for Structural Bioinformatics (RCSB) Protein Data Bank (PDB) (https://www.rcsb.org). The structure of AG1529 was drawn and transformed into 3D structure with Marvin Sketch from ChemAxon (https://chemaxon.com/). Capsaicin (PubChem ID: 1548943) and capsazepine (PubChem ID: 2733484) structures were obtained from the National Center for Biotechnology Information (NCBI) PubChem database (http://www.ncbi.nlm.nih.gov/pccompound). The global docking procedure was accomplished with AutoDock 4^46^ implemented in Yasara^47^, in which a total of 800 flexible docking runs were set and clustered around the putative binding sites. The program then performed a simulated annealing optimization of the complexes, which moved the structure to a nearby stable energy minimum, by using the implemented Assisted Model Building with Energy Refinement (AMBER03) force field^48^. The Yasara pH command was set to 7.0, to ensure that molecules preserve their pH dependency of bond orders and protonation patterns. The best binding energy complex in each cluster was stored, analyzed, and used to select the best orientation of the interacting partners. The more positive the interaction energy, the more favorable the interaction is. To further explore the best binding site for each molecule a focalized docking assay with a total of 150 flexible dockings was performed in a limited area of 22:30:25 Å (x:y:z) around the capsaicin binding site centered in the global docking conformation that adopted each molecule in this particular site. Figures were drawn with open source PyMol (The PyMOL Molecular Graphics System, Version 1.8 Schrödinger, LLC, at http://www.pymol.org/).

### Statistical analysis

All data are expressed as mean ± SEM. The number of replicates is indicated in the figure legends. In vitro data were statistically analyzed using One-Way ANOVA followed by the Bonferroni post-hoc test of multiple comparison as indicated, or unpaired, two-tail Student t-test for some experiments also indicated. For One-Way ANOVA we report the F (DFn, DFd) and the P value, along with the p values derived from the Bonferroni post-hoc test. p-value for all the analyzes was set at 0.05, and the value obtained is reported. For in vivo experiments, the number of animals used was n = 4–7 as indicated.
